# Influence of Irradiation Temperature on Oxidative and Network Properties of X-Ray Cross-Linked Vitamin E Stabilized UHMWPE for Hip Arthroplasty

**DOI:** 10.1155/2020/2568428

**Published:** 2020-03-23

**Authors:** M. A. Mulliez, C. Schilling, T. M. Grupp

**Affiliations:** ^1^Aesculap AG, Research & Development, 78532 Tuttlingen, Germany; ^2^Ludwig Maximilians University, Department of Orthopaedics Surgery, Physical Medicine & Rehabilitation, Campus Grosshadern, 81377 Munich, Germany

## Abstract

Previous studies have shown that increased cross-link density, reduced free radicals, and increased antioxidant grafting resulting from electron-beam irradiation at elevated temperatures improved the wear performance and the oxidative stability of vitamin E blended UHMWPE. The current study explores the impact of elevated irradiation temperature on vitamin E blended UHMWPE using X-ray. We hypothesize that the effects of temperature would be similar to those observed after electron-beam irradiation due to the relatively high dose rate of X-rays. Two X-ray doses of 80 and 100 kGy and two irradiation temperatures, that is, room temperature and 100°C were considered. The reference was Vitelene®, a vitamin E stabilized polyethylene cross-linked with 80 kGy by e-beam at 100°C. Oxidation index and oxidation induction time, as well as cross-link density, gel fraction, and *trans*-vinylene index, were determined, as the oxidative and network properties are decisive for the long-term implant performance. Gel fraction and oxidation induction time were significantly improved subsequently to warm irradiation in comparison with the material irradiated at room temperature. In conclusion, X-ray irradiation at elevated temperatures resulted in an increase of cross-linking and oxidative resistance of vitamin E stabilized polyethylene comparable to those of e-beam irradiated UHMWPE.

## 1. Introduction

It is clinically established that the creation of a three-dimensional polymer chain network by means of cross-linking increases the wear resistance of polyethylene orthopedic implants considerably [1, 2]. Radiation cross-linking is characterized by convenience and efficiency in comparison to chemical alternatives. It has the major advantage that no chemical adjuvant like a catalyst or special environmental factors like pressure or temperature is needed to perform the reaction: it makes chemical reactions in a solid polymer at room temperature possible. As a pure physical process, it does not lead to residues of alien substances with toxic potential, which is essential when using it for implantable medical devices [3–6].

During irradiation at high to very high radiation doses (50–10,000 kGy), the polyethylene undergoes two types of modification: cross-linking corresponding to the creation of a spatial network through the connection of macromolecular chains to each other [7, 8] and detrimental changes: scissions [2]. In polyethylene, cross-linking prevails over scissioning [9]. Energy source, dose rate, absorbed dose, processing conditions “prior to, during, and after” irradiation, as well as temperature, environment (air, inert, vacuum), time, and the presence of additives in the polymer influence the recombination ratio cross-linking/chain scission [5, 7, 10–12].

Electron-beam (e-beam) exhibits a reduced penetration capacity, whereas Gamma ray exhibits a low dose rate [13, 14]. Ionization radiation in air at low dose rate requires longer treatment time. The greater exposition to air can facilitate the diffusion of oxygen in the polymer and stimulate the oxidative scission reaction [5, 6, 15, 16]. The proportion of scission to cross-linking rises and deterioration of the material properties is intensified [3]. A higher dose rate of irradiation preserves the material for oxidative degradation [17] and promotes cross-linking over chain scission [18]. This time dependence of oxygen diffusion during irradiation exists at room temperature but is even more relevant at elevated temperature, as heat promotes oxygen diffusion.

Based on these insights the authors used X-ray irradiation as a new approach to overcome the described limitations. Shortly after X-rays and their ability to penetrate matter have been discovered by W. C. Röntgen in 1895 [19], their effect on biological, physical, and chemical material properties was identified. One of the first medical applications was the X-ray tubes for blood irradiation. The dose needed for diagnostic and therapeutic purposes amounts 0.01–10 Gy, whereas industrial applications require up to multiple kilo-grays [19, 20]. With its high penetration depth and a moderate dose rate, X-ray offers optimized processing parameters which allow for warm irradiation and for preserving from early oxidation as e-beam does.

The objective of this study was to evaluate the influence of the irradiation temperature on the network properties (*trans-vinylene* index, gel content, degree of cross-linking) and the oxidative behavior virgin and after accelerated aging (oxidation and oxidation resistance via oxidation induction time) of vitamin E stabilized UHMWPE (ultrahigh molecular weight polyethylene) cross-linked using X-rays as a new irradiation source in comparison with e-beam. The hypothesis was that an elevation of the irradiation temperature would have similar effects on the aforementioned material properties after cross-linking whether by X-ray or by state-of-the-art e-beam irradiation.

## 2. Materials and Methods

### 2.1. Materials

The specimens were made of GUR® 1020 blended with 0.1 weight percent vitamin E. After consolidation and annealing, 60 × 60 × 200 mm bars were cut from the sheet and submitted to X-ray radiation cross-linking. The material was split into four groups distinguished by absorbed dose and irradiation temperature, respectively, as follows: 80 kGy, room temperature (RT) “X (80 kGy)-RT;” 100 kGy, room temperature “X (100 kGy)-RT;” 80 kGy, 100°C “X (80 kGy)-warm;” 100 kGy, 100°C “X (100 kGy)-warm.” Prior to irradiation the bars were introduced in an insulation box and preheated below the melting point in an oven. Temperature and cooling kinetics were determined on the occasion of preliminary tests to ensure a minimum of 100°C during the complete X-ray irradiation process. The test bars were cross-linked by means of X-ray with a double-sided irradiation of each 20 kGy at a dose rate of 0.26 kGy/s. The X (80 kGy) specimens were subjected to four runs, the X (100 kGy) to five runs. The absorbed dose was determined during a pretest thanks to alanine dosimeters.

Vitelene® (Aesculap AG, Tuttlingen, Germany), a highly cross-linked (electron-beam, 100°C, 80 kGy with a 10 MeV-Rhodotron®), vitamin E (0.1%) blended polyethylene available on the market as part of a hip joint prosthesis was taken as reference.

No further postirradiation thermal treatment was performed.

### 2.2. Methods

#### 2.2.1. Oxidative Characterization

To determine the oxidative properties of X-ray and e-beam cross-linked vitamin E stabilized polyethylene, the Oxidation Index (OI) and the Oxidation Induction Time (OIT) were determined. Accelerated aging according to ASTM F2003-02(2015) was performed in order to compare the oxidation resistance and oxidation potential of the different materials.

The OI was measured with a Fourier Transform Infrared (FTIR) spectrometer by dividing the area of the carbonyl absorptions (>C=O) centered near 1720 cm^−1^ by the area of the normalization peak (C-H absorptions) centered near 1370 cm^−1^ according to ASTM F2102-17.

X-ray specimens were obtained from bulk material. Slices were cut from the surface at depths 0, 100, 200, and 500 *μ*m from two opposite faces. We limited the measurement to 500 *μ*m because in agreement with our experience no significant oxidation could be observed for vitamin E blended polyethylene over this depth even after accelerated aging. Vitelene® serial implants were halved and sliced. A line scan was recorded across the sample towards the surface at 100 *μ*m increments to a depth of 3 mm. The maximum oxidation index was collected.

As OIT correlates with antioxidant efficacy, it was used to compare the oxidative resistance of the tested materials. The OIT measurements were performed with a differential scanning calorimeter according to ASTM D3895-14. The polyethylene samples were taken from the surface and were heated under a nitrogen atmosphere up to 200°C. After that, the atmosphere was switched to oxygen. The time until the exothermal degradation began was recorded.

#### 2.2.2. Network Characterization

The following analyses provide information about radiolytic events occurring during the irradiation of polyethylene like the formation of *trans*-vinylene unsaturations and cross-linkings.

At first, the *trans*-vinylene index (TVI) was determined with a FTIR spectrometer by calculating the ratio of the area of the absorption peak centered on 965 cm^−1^ to the area of the normalization peak centered on 1370 cm^−1^ according to ASTM F2381-10. The yields of *trans*-vinylene radiolytic unsaturations are proportional to the number of cross-links formed [21, 22]. The TVI is a reliable indicator of dose level [12, 23].

Second, the gel and soluble fraction of each material were determined gravimetrically according to ASTM D2765-16. When submitted to radiation emanating from a reactor, polyethylene forms an insoluble gel [13, 24] which gives information about the fraction of long molecules which are entangled and/or cross-linked [12]. During the extraction in xylene the soluble part of the network is extracted whereas the cross-linked is not. The specimens were weighed (*m*_*i*_), immersed and refluxed for 12 hours in boiling xylene. Afterwards, they were dried and weighed (*m*_*f*_) another time. The soluble fraction (*w*_soluble_) in % mass was calculated as follows:

(1) $$\begin{array}{c} w_{\text{soluble}}=100\times \frac{\left(m_{i}-m_{f}\right)}{m_{i}}. \end{array}$$

The gel fraction (*w*_Gel_) in % mass was calculated as follows:

(2) $$\begin{array}{c} w_{\text{Gel}}=100-w_{\text{soluble}}. \end{array}$$

Third, the degree of cross-linking or the number of moles of cross-links per unit volume has been determined as an instructive method in understanding the relative cross-link density between different materials [25]. Direct determination is impossible because it cannot be distinguished between the physical and the chemical cross-links [12, 26]. The swell ratio (*ρ*) was determined gravimetrically from the absorbed xylene weight divided by its density (0.75 g/cm³) after immersion at 130°C for 2 hours.

(3) $$\begin{array}{c} \rho =\frac{\left(V_{s}+V_{x}\right)}{V_{s}}, \end{array}$$

with *V*_*s*_ = initial volume of the specimen as the result of its initial weight divided by its density *d* assumed to be 0.935 g/cm³, and *V*_*x*_ = volume of the absorbed xylene as the difference between the final xylene-swollen and the initial weight of the specimen divided by the density of xylene (0.75 g/cm³).

The cross-link density (*v*_*d*_) was indirectly calculated based on the determination of the swell ratio and the Flory network theory [27]

(4) $$\begin{array}{c} v_{d}=-\frac{\ln\left(1-\left(1/\rho \right)\right)+\left(1/\rho \right)+\left(\left(1/3\right)+\left(5/9\rho \right)\right)/\rho^{2}}{136\left(\rho^{-1/3}-\left(1/2\rho \right)\right)}. \end{array}$$

The molecular mass between cross-linking (Mw) was determined as follows:

(5) $$\begin{array}{c} \text{Mw}=\frac{d}{v_{d}}. \end{array}$$

For every test *n* was ≥3, except for the gel fraction *n* = 1.

### 2.3. Statistics

To differentiate the network and oxidative characteristics between the five material groups, an analysis of variance ANOVA was carried out (*p* < 0.05) followed by a post hoc test (Tukey's HSD-Test, *p* < 0.05).

Prior to analysis, the normal distribution (p-p plots) and the homogeneity of variance (Levene Test) were verified (Statistica R13, TIBCO Software Inc.). A *p* value of less than 0.05 was considered significant.

## 3. Results

### 3.1. Oxidative Characterization

#### 3.1.1. Oxidation Index (Figure 1)

The FTIR spectra revealed low oxidation indices (<0.20), less oxidation for warm irradiated polyethylene than for the material processed at room temperature (*p* < 0.001). The X-ray warm irradiated materials showed little to no significant oxidation (their OI level was very close to the limit of quantification 0.025). A light increased oxidation could be observed between 80 and 100 kGy at room temperature, but it was not found to be statistically significant (*p*=0.180 virgin and *p*=0.227 aged). The OI level was equivalent before and after accelerated aging. No significant difference between e-beam 80 kGy warm (Vitelene®) and X-ray cross-linking 80 kGy warm was seen (*p*=1).

**Figure 2:**
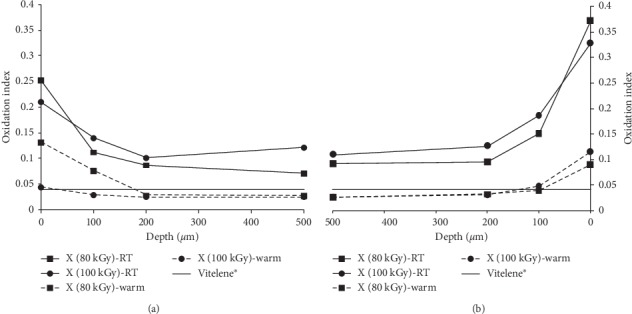
Surface oxidation of the X-ray cross-linked UHMWPE feedstock and of the Vitelene® implants, both virgin. (a) Subsurface OI measured from one side of the test bar. (b) Subsurface OI measured from the opposite side of the test bar. LOQ: 0.025.

The FTIR analysis of the feedstock surface exhibited an oxidation index up to four times higher than that of the subsurface (Figure 2).

#### 3.1.2. Oxidation Induction Time

First of all, relatively low values for irradiation at room temperature and to some extent a large standard deviation of up to 80% were observed. The reason for these findings might be the fact that most of the samples for the measurement were taken out of the surface of the feedstock and others out of a cut face. The surface has been subjected to high thermal and mechanical stresses during manufacturing what was responsible for increased degradation (Table 1).

Although the temperature dependence could not be always statistically confirmed (virgin: *p*=0.003 for 80 kGy, *p*=0.168 for 100 kGy, aged: *p*=0.001 for 80 kGy, *p*=0.534 for 100 kGy, between room temperature and warm irradiation, respectively), the positive influence of heat supply during irradiation processing was obvious.

No dose-related significant difference could be observed neither for irradiation at room temperature nor for warm irradiation (*p* > 0.1).

### 3.2. Network Characterization

Table 2 gives a summary of the results of the network characterization.

#### 3.2.1. *trans*-Vinylene Index

**Figure 3:**
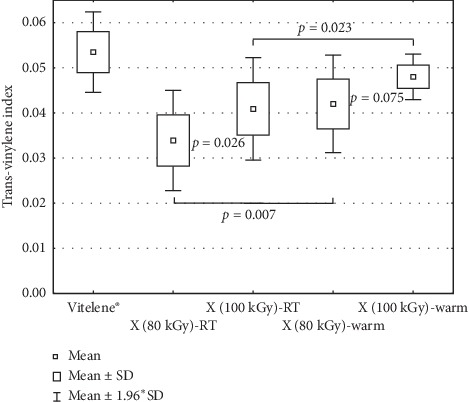
*trans*-vinylene unsaturations of Vitelene®, X (80 kGy)-RT, X (100 kGy)-RT, X (80 kGy)-warm, and X (100 kGy)-warm.

Both the irradiation temperature and the dose were found to affect the amount of *trans*-vinylene unsaturations with higher TVI for warm irradiation and increased dose. UHMWPE irradiated with e-beam exhibited higher TVI as well (Figure 3, Table 2).

#### 3.2.2. Gel Fraction

The data in Table 2 show that the difference between samples irradiated at 80 kGy and 100 kGy was almost negligible concerning the solubility measurement. In contrast, enhancing irradiation temperature led to a higher insoluble fraction of the polymer.

No difference was seen between e-beam and X-ray.

#### 3.2.3. Degree of Cross-Linking

Except between Vitelene® and X (80 kGy)-warm (*p* = 0.015) the differences between cross-link densities were not statistically significant (*p* = 0.143 to 0.999). However, a slight increase could be observed with a higher dose. Neither the irradiation temperature nor the radiation source seemed to substantially affect the cross-link density.

## 4. Discussion

The objective of this study was to evaluate the influence of the irradiation temperature on the network properties (TVI, gel content, degree of cross-linking) and the oxidative behavior virgin and after accelerated aging (oxidation and oxidation resistance via OIT) of vitamin E stabilized UHMWPE cross-linked using X-rays as a new irradiation source in comparison with e-beam. The hypothesis was that an elevation of the irradiation temperature would have similar effects on the aforementioned properties after cross-linking whether by X-ray or by state-of-the-art e-beam irradiation.

### 4.1. Limitations

This work is subjected to some limitations.

First, we investigated only 2 irradiation doses in the same clinically relevant range. For this reason, the real influence of the dose could not be systematically analyzed.

Second, our in vitro aging model did not reproduce all the mechanisms involved in initiating and propagating oxidation in vivo like mechanical stress, cyclic loading, and influence of lipids (squalene) [28–34].

Third, cross-linking density was determined below the melt temperature. The crystalline phase did not vanish completely. Therefore, not only the persisting cross-links in the amorphous phase were considered but also other physical bonds like entanglements and crystals. Rheological characteristics in the melt could give more information about the real cross-link density [12].

As accentuated by Premnath et al. [35], the determination of the cross-link density according to Flory–Rehner expression for swollen cross-linked networks is not possible since the irradiation cross-linking carried out in the solid state does not take place homogeneously but preferentially in the amorphous region [7, 18]. Therefore, our measurement serves only as a comparison of the different materials.

Fourth, since the oxidative degradation of polyethylene is mostly due to long-lived radiation-induced free radicals particularly those trapped in the crystalline phase, an Electron Spin Resonance analysis could help predict the oxidation kinetic. At last, the FTIR determination of hydroperoxides, which are intermediate oxidation products, could have been carried out as a measure of oxidation potential [36].

The focus was the evaluation of X-rays versus e-beam at elevated temperatures to cross-link UHMWPE implant material. Further investigations would be of scientific interest but beyond the scope of the current study.

### 4.2. Oxidative Characterization

#### 4.2.1. Oxidation Index

The OI levels were in the same range (<0.2) as reported in the literature for XLPE stabilized with vitamin E [33, 37, 38] and far below 1 which was considered to be the limit associated with the degradation of mechanical properties for conventional polyethylene [39–42]. However, to what extent this threshold applies for highly cross-linked polyethylene is unknown.

The FTIR analysis confirmed our hypothesis: OI of Vitelene® (*β*-80 kGy, warm) and X (80 kGy)-warm were found to be equivalent (*p* = 1). The presence of vitamin E and the elevated irradiation temperature may explain why in contradiction to Wannomae and Muratoglu [43] no detrimental effect of X-ray irradiation on the oxidation resistance of the material was observed. Elevation of the irradiation temperature was shown to be useful against oxidative degradation. Several reasons are possible: the heat increases the mobility of the chains and promotes the free radical decay [28, 29] and/or the heat may favor the chemical bonding of the vitamin E to the polyethylene chains during irradiation which prevents loss of oxidation resistance [28]. The similar OI before and after 2 weeks accelerated aging is attributed to the presence of the antioxidant vitamin E. The accelerated aging according to ASTM F2003 for 2 weeks was not able to generate a difference between the materials.

The high oxidation level (up to 0.6) on the surface layer of the X-ray irradiated feedstocks in air was attributed to immediate oxidative degradation, also in the presence of vitamin E [12, 18, 33, 44, 45]. Oxygen is able to diffuse within about 0.5 to 2 mm the polyethylene surface [21, 31]. Dissolved oxygen reacts with the free radicals even in the presence of a small amount of antioxidant (0.05–0.1%) [12, 28, 33, 34, 46–49]. Furthermore, the degradation of vitamin E at higher temperatures can occur [50] and the low vitamin E concentration could not prevent completely the surface oxidation during processing. The oxidation cascade is hindered as vitamin E gives hydrogen to the free radicals and the further oxidation of the material is prevented.

In contrast to Oral et al. [30] and Slouf et al. [18], no raised oxidation with increased dose could be noticed. This was probably due to the relatively small dose difference of 20 kGy.

As oxidative degradation remains ongoing in vivo and in the long-term [15, 29, 30, 33, 39, 51–53], a manufacturing method that allows for optimal preservation of the antioxidant is highly desirable.

#### 4.2.2. Oxidation Induction Time

OIT was determined to compare the antioxidative potential of the materials.

The values were in the same range as mentioned in the literature [54]. The large variance did not permit any reliable conclusions about the role of the dose. However, the beneficial effect of irradiation at increased temperature was confirmed. Heat improves vitamin E preservation and grafting during irradiation. This would probably result in increased long-term stability [29, 50, 52, 53, 55]. Furthermore, a stabilizing activity of the α-tocopherol derivatives could amplify the effect [56]. In contrast to the findings of Wannomae et al. no deleterious effect of the X-ray irradiation on the antioxidative potency was asserted [43]. Although the reliability of this measurement is controversially discussed particularly for low OIT values (<15 minutes) [57], this method is described to be helpful for assessing and ranking the antioxidative potential of antioxidant-containing UHMWPEs [54, 58]. As the test is conducted under very harsh conditions which absolutely do not correspond to in vivo aging, it serves exclusively the material comparison and a correlation with the clinical behavior remains to be established.

### 4.3. Network Characterization

#### 4.3.1. *trans*-Vinylene Index

The yield of *trans*-vinylene unsaturations was higher for warm irradiation than for irradiation at room temperature. It can be interpreted such that the temperature increases the radiolytic reactions kinetics [23, 59, 60].

Furthermore, more *trans*-vinylene units were formed at 100 kGy than at 80 kGy confirming their augmentation with increasing the absorbed dose [18, 33, 60–67].

We observed a tendentially higher response for e-beam (on average, because of its high dose uniformity ratio) than for X-ray. The irradiation source affects the reaction kinetics as well as the dose rate, the absorbed dose, and the temperature [33, 65].

#### 4.3.2. Gel Fraction

The extraction test verified that all the materials were highly cross-linked. As a gel fraction of 75% is typically reached after sterilization with 25–35 kGy [54] higher doses lead systematically to gel fractions between 90% and 100% making the differentiation of the materials based on this parameter difficult. However, some tendencies could be seen.

The gel fraction up to 99% for the warm irradiated groups indicated that only little chain scissioning occurred or that the radicals created during irradiation recombined in a cage reaction during swelling in xylene [68]. The higher gel content at elevated temperature can be explained by the reduced mobility of vitamin E due to greater grafting. This reduces the reaction competition and promotes the recombination of the free radicals to form cross-links [53]. In contrast to the literature, no increase of gel content with dose was observed [69]. Vitelene® and X (80 kGy)-warm exhibited equivalent gel content supporting our hypothesis.

#### 4.3.3. Cross-Link Density

In opposition to the literature [23, 26, 53, 59, 61, 64, 70–73], no significant influence of dose and temperature (*p* > 0.05) was noticed. One reason for this discrepancy might be the error in the measurement of the swollen mass which increases as the swell ratio decreases [35]. Another reason might be the fact that the cross-link density was determined at 130°C, whereby the melting range of UHMWPE extends from 50°C to 160°C [54]. In this way, much more short chains and entanglements still remain and bias the determination of the cross-link density.

As the cross-link density is directly related to the wear behavior of UHMWPE [70, 74], particular attention should be paid to the result of the oncoming wear simulation tests.

## 5. Conclusion

It is currently accepted that the combined advantages of cross-linking and stabilization with a suitable antioxidant lead to an extension of the lifetime of orthopedic implants. However, oxidation is initiated not only as a result of irradiation but is induced in vivo as well. Taking into consideration that vitamin E has a limited potency, it is worthwhile to prevent its degradation during the manufacturing process in order to prolong its efficacy in vivo and in this way further increase the longevity of the prosthesis.

Our study confirmed the positive effect of the elevated irradiation temperature on the radiation cross-linking and on the oxidation resistance of vitamin E stabilized UHMWPE, independently of the irradiation source e-beam or X-ray.

## Figures and Tables

**Figure 1:**
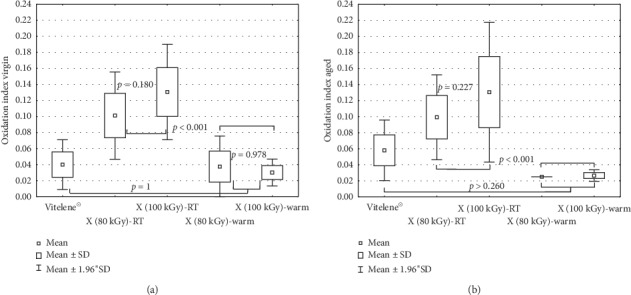
Oxidation index of Vitelene® implants, X (80 kGy)-RT, X (100 kGy)-RT, X (80 kGy)-warm, and X (100 kGy)-warm, virgin (a) and after accelerated aging (b) according to ASTM F2003 for 2 weeks after elimination of the first 100 *μ*m of the feedstock. Limit of quantification (LOQ): 0.025.

**Table 1:** OIT of Vitelene® implants, X (80 kGy)-RT, X (100 kGy)-RT, X (80 kGy)-warm, and X (100 kGy)-warm, virgin and after accelerated aging according to ASTM F2003 for 2 weeks.

	OIT-virgin (min)	OIT-aged (min)
Vitelene®	11.3 ± 1.8	10.9 ± 1.2
X (80 kGy)-RT	6.2 ± 3.1	3.7 ± 2.9
X (100 kGy)-RT	5.6 ± 2.9	5.7 ± 0.0
X (80 kGy)-warm	18.8 ± 0.4	12.8 ± 0.6
X (100 kGy)-warm	11.8 ± 5.9	8.4 ± 3.7

**Table 2:** Network properties of Vitelene®, X (80 kGy)-RT, X (100 kGy)-RT, X (80 kGy)-warm, and X (100 kGy)-warm.

	TVI	Gel content (%)	Cross-link density (mol/dm³)	Mw between cross-links (g/mol)
Vitelene®	0.053 ± 0.004	99	0.195 ± 0.007	4800 ± 173
X (80 kGy)-RT	0.034 ± 0.006	93	0.179 ± 0.006	5222 ± 190
X (100 kGy)-RT	0.041 ± 0.006	94	0.183 ± 0.012	5133 ± 355
X (80 kGy)-warm	0.042 ± 0.006	99	0.172 ± 0.005	5450 ± 157
X (100 kGy)-warm	0.048 ± 0.003	99	0.180 ± 0.009	5213 ± 257

## Data Availability

The results of the measurements or the data used to support the findings of this study are included within the article (Figures 1–3 and Tables 1 and 2).

## Additional Points

*Statement of Significance*. Two important topics when considering the clinical relevance of the study are the cross-link density and the long-term oxidative stability. The cross-link density is directly related to the wear resistance. Oxidation promotes chain scission and reduces the yield of cross-linking. Although its clinical effects and impact on implant revision are still unclear especially for cross-linked UHMWPE, the resistance to oxidation allows the wear and mechanical properties to be maintained in the long run. Therefore, it is necessary to pay particular attention to the influence of the irradiation conditions of X-ray as a quite new cross-linking method on the aforementioned material properties.
